# *N*-Cyclo­hexyl-2-(5-meth­oxy-1*H*-indol-3-yl)-2-oxoacetamide

**DOI:** 10.1107/S1600536810054656

**Published:** 2011-01-08

**Authors:** Jing Liu, Yong-Feng Liu, Shi Zhang, Ying Gao, Hong Chen

**Affiliations:** a School of Pharmacy, Tianjin Medical University, Tianjin 300070, People’s Republic of China; b Room of Pharmacognosy, Medical College of Chinese People’s Armed Police Forces, Tianjin 300162, People’s Republic of China; c Tianjin Key Laboratory for Biomarkers of Occupational and Environmental Hazards, Tianjin 300162, People’s Republic of China

## Abstract

In the title compound, C_17_H_20_N_2_O_3_, the cyclo­hexane ring adopts a chair conformation. In the crystal, inter­molecular N—H⋯O hydrogen bonds link the mol­ecules into layers parallel to the *ac* plane.

## Related literature

For the biological activity of indole derivatives, see: Souli *et al.* (2008 ▶); Chai *et al.* (2006 ▶); Radwan *et al.* (2007 ▶); Karthikeyan *et al.*, (2009 ▶). For details of the synthesis, see: Bacher *et al.* (2001 ▶). For similar structures, see: Feng *et al.* (2008 ▶); Sonar *et al.* (2006 ▶).
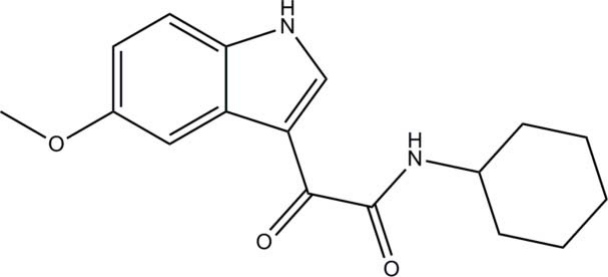


## Experimental

### 

#### Crystal data

C_17_H_20_N_2_O_3_
*M* *_r_* = 300.35
Monoclinic, 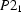

*a* = 5.083 (3) Å
*b* = 27.336 (13) Å
*c* = 5.220 (3) Å
β = 91.977 (12)°
*V* = 724.9 (6) Å^3^
*Z* = 2
Mo *K*α radiation
μ = 0.10 mm^−1^
*T* = 113 K
0.20 × 0.18 × 0.10 mm

#### Data collection

Rigaku Saturn CCD area-detector diffractometer
Absorption correction: multi-scan (*CrystalClear*; Rigaku, 2005 ▶) *T*_min_ = 0.981, *T*_max_ = 0.991
7342 measured reflections
1764 independent reflections
1569 reflections with *I* > 2σ(*I*)
*R*_int_ = 0.042

#### Refinement

*R*[*F* ^2^ > 2σ(*F* ^2^)] = 0.035
*wR*(*F* ^2^) = 0.076
*S* = 1.02
1764 reflections
208 parameters
1 restraint
H atoms treated by a mixture of independent and constrained refinement
Δρ_max_ = 0.23 e Å^−3^
Δρ_min_ = −0.18 e Å^−3^

### 

Data collection: *CrystalClear* (Rigaku, 2005 ▶); cell refinement: *CrystalClear*; data reduction: *CrystalClear*; program(s) used to solve structure: *SHELXTL* (Sheldrick, 2008 ▶); program(s) used to refine structure: *SHELXTL*; molecular graphics: *XP* in *SHELXTL*; software used to prepare material for publication: *SHELXTL*.

## Supplementary Material

Crystal structure: contains datablocks I, global. DOI: 10.1107/S1600536810054656/cv5025sup1.cif

Structure factors: contains datablocks I. DOI: 10.1107/S1600536810054656/cv5025Isup2.hkl

Additional supplementary materials:  crystallographic information; 3D view; checkCIF report

## Figures and Tables

**Table 1:** Hydrogen-bond geometry (Å, °)

*D*—H⋯*A*	*D*—H	H⋯*A*	*D*⋯*A*	*D*—H⋯*A*
N1—H1⋯O2^i^	0.96 (3)	1.90 (3)	2.840 (2)	164 (3)
N2—H2⋯O3^ii^	0.88 (3)	2.09 (3)	2.926 (3)	160 (3)

Symmetry codes: (i) 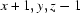; (ii) 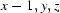.

**Fig. 1.**
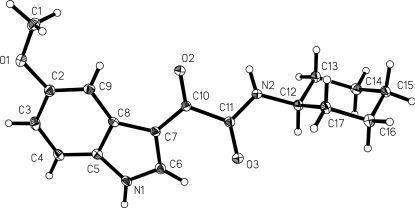
View of the title compound, with displacement ellipsoids drawn at the 40% probability level.

## supplementary crystallographic information

### Comment

Indole and their derivatives are well known as substances exhibiting various biological activiies, such as anti-cancer (Souli *et al.*, 2008), anti-viral (Chai *et al.*, 2006), anti-tubercular (Karthikeyan *et al.*, 2009) and anti-inflammatory (Radwan *et al.*, 2007). In our search for new indole derivatives with improved activities, we have synthesized the title compound, (I). Here we report its crystal structure.

In (I) (Fig. 1), all bond lengths and angles are normal and in a good agreement with those reported previously (Feng *et al.*, 2008; Sonar *et al.*, 2006). The cyclohexane ring (C12—C17) adopts a chair conformation. In the crystal structure, intermolecular N—H···O hydrogen bonds (Table 1) link the molecules into layers parallel to the *ac* plane.

### Experimental

The target compound was synthesized by two steps. Oxalyl chloride was added dropwise to a solution of 5-methoxy-indole in dry ether, the crude product 5-methoxyindol-3-yl-glyoxyl chloride, cyclohexylamine, two drops of triethylamine in dry dichloromethane. The reaction mixture was washed with water and dried over MgSO4 and concentrated *in vacuo* (Bacher *et al.*, 2001). The residue was resolved in a methanol solution. Slow evaporation over two weeks at room temperature gave light-yellow crystals suitable for X-ray analysis.

### Refinement

C-bound H atoms were geometrically positioned (C—H = 0.95–1.00 Å), and refined as riding, with *U*_iso_(H) = 1.2-1.5 *U*_eq_(C). Amino H atoms were located on a difference map and refined isotropically.

### Crystal data

**Table:**

C_17_H_20_N_2_O_3_	*F*(000) = 320
*M**_r_* = 300.35	*D*_x_ = 1.376 Mg m^−^^3^
Monoclinic, *P*2_1_	Mo *K*α radiation, λ = 0.71073 Å
Hall symbol: P 2yb	Cell parameters from 2636 reflections
*a* = 5.083 (3) Å	θ = 1.5–27.9°
*b* = 27.336 (13) Å	µ = 0.10 mm^−^^1^
*c* = 5.220 (3) Å	*T* = 113 K
β = 91.977 (12)°	Prism, colourless
*V* = 724.9 (6) Å^3^	0.20 × 0.18 × 0.10 mm
*Z* = 2	

### Data collection

**Table:**

Rigaku Saturn CCD area-detector diffractometer	1764 independent reflections
Radiation source: rotating anode	1569 reflections with *I* > 2σ(*I*)
multilayer	*R*_int_ = 0.042
Detector resolution: 14.63 pixels mm^-1^	θ_max_ = 27.9°, θ_min_ = 1.5°
ω and φ scans	*h* = −6→6
Absorption correction: multi-scan (*CrystalClear*; Rigaku, 2005)	*k* = −36→31
*T*_min_ = 0.981, *T*_max_ = 0.991	*l* = −6→6
7342 measured reflections	

### Refinement

**Table:**

Refinement on *F*^2^	Primary atom site location: structure-invariant direct methods
Least-squares matrix: full	Secondary atom site location: difference Fourier map
*R*[*F*^2^ > 2σ(*F*^2^)] = 0.035	Hydrogen site location: inferred from neighbouring sites
*wR*(*F*^2^) = 0.076	H atoms treated by a mixture of independent and constrained refinement
*S* = 1.02	*w* = 1/[σ^2^(*F*_o_^2^) + (0.0439*P*)^2^] where *P* = (*F*_o_^2^ + 2*F*_c_^2^)/3
1764 reflections	(Δ/σ)_max_ = 0.003
208 parameters	Δρ_max_ = 0.23 e Å^−^^3^
1 restraint	Δρ_min_ = −0.18 e Å^−^^3^

### Special details

**Table:**

Geometry. All e.s.d.'s (except the e.s.d. in the dihedral angle between two l.s. planes) are estimated using the full covariance matrix. The cell e.s.d.'s are taken into account individually in the estimation of e.s.d.'s in distances, angles and torsion angles; correlations between e.s.d.'s in cell parameters are only used when they are defined by crystal symmetry. An approximate (isotropic) treatment of cell e.s.d.'s is used for estimating e.s.d.'s involving l.s. planes.
Refinement. Refinement of *F*^2^ against ALL reflections. The weighted *R*-factor *wR* and goodness of fit *S* are based on *F*^2^, conventional *R*-factors *R* are based on *F*, with *F* set to zero for negative *F*^2^. The threshold expression of *F*^2^ > σ(*F*^2^) is used only for calculating *R*-factors(gt) *etc*. and is not relevant to the choice of reflections for refinement. *R*-factors based on *F*^2^ are statistically about twice as large as those based on *F*, and *R*- factors based on ALL data will be even larger. Along with the meaningless absolute structure parameter value (and s.u. value) obtained from any refinement with Friedel pairs, as justification of the merging of Friedel-pair data.

### Fractional atomic coordinates and isotropic or equivalent isotropic displacement parameters (Å^2^)

**Table:**

	*x*	*y*	*z*	*U*_iso_*/*U*_eq_	
O1	−0.1844 (3)	0.03318 (6)	0.3071 (3)	0.0242 (4)	
O2	−0.1506 (3)	0.20016 (5)	0.8241 (3)	0.0181 (3)	
O3	0.3519 (3)	0.28545 (6)	0.8311 (3)	0.0209 (4)	
N1	0.4663 (4)	0.19176 (7)	0.2050 (3)	0.0175 (4)	
N2	−0.0791 (4)	0.29907 (6)	0.8997 (3)	0.0167 (4)	
C1	−0.3621 (4)	0.03287 (9)	0.5127 (5)	0.0241 (5)	
H1A	−0.2627	0.0360	0.6761	0.036*	
H1B	−0.4607	0.0021	0.5106	0.036*	
H1C	−0.4851	0.0603	0.4927	0.036*	
C2	−0.0281 (4)	0.07411 (8)	0.2854 (4)	0.0191 (5)	
C3	0.1505 (4)	0.07156 (8)	0.0851 (4)	0.0207 (5)	
H3	0.1508	0.0435	−0.0223	0.025*	
C4	0.3242 (4)	0.10905 (8)	0.0430 (4)	0.0185 (4)	
H4	0.4446	0.1075	−0.0920	0.022*	
C5	0.3169 (4)	0.14924 (7)	0.2052 (4)	0.0158 (4)	
C6	0.3895 (4)	0.22142 (8)	0.3936 (4)	0.0182 (4)	
H6	0.4632	0.2526	0.4319	0.022*	
C7	0.1850 (4)	0.19958 (8)	0.5251 (4)	0.0147 (4)	
C8	0.1372 (4)	0.15268 (8)	0.4031 (4)	0.0153 (4)	
C9	−0.0386 (4)	0.11418 (8)	0.4453 (4)	0.0170 (4)	
H9	−0.1603	0.1156	0.5792	0.020*	
C10	0.0403 (4)	0.22048 (7)	0.7283 (4)	0.0150 (4)	
C11	0.1203 (4)	0.27147 (7)	0.8258 (4)	0.0154 (4)	
C12	−0.0360 (4)	0.35000 (7)	0.9780 (4)	0.0168 (4)	
H12	0.1085	0.3636	0.8739	0.020*	
C13	−0.2834 (4)	0.38039 (8)	0.9209 (4)	0.0187 (4)	
H13A	−0.4302	0.3675	1.0208	0.022*	
H13B	−0.3340	0.3777	0.7366	0.022*	
C14	−0.2356 (5)	0.43413 (8)	0.9899 (4)	0.0194 (5)	
H14A	−0.4000	0.4529	0.9587	0.023*	
H14B	−0.0995	0.4478	0.8793	0.023*	
C15	−0.1453 (4)	0.43918 (8)	1.2721 (4)	0.0184 (4)	
H15A	−0.1048	0.4739	1.3106	0.022*	
H15B	−0.2888	0.4287	1.3832	0.022*	
C16	0.0984 (4)	0.40803 (8)	1.3293 (4)	0.0205 (5)	
H16A	0.1486	0.4106	1.5137	0.025*	
H16B	0.2466	0.4206	1.2301	0.025*	
C17	0.0493 (4)	0.35425 (8)	1.2602 (4)	0.0185 (5)	
H17A	0.2123	0.3351	1.2934	0.022*	
H17B	−0.0897	0.3408	1.3682	0.022*	
H1	0.603 (6)	0.2006 (11)	0.090 (6)	0.042 (8)*	
H2	−0.240 (5)	0.2875 (10)	0.891 (5)	0.027 (7)*	

### Atomic displacement parameters (Å^2^)

**Table:**

	*U*^11^	*U*^22^	*U*^33^	*U*^12^	*U*^13^	*U*^23^
O1	0.0251 (8)	0.0167 (8)	0.0310 (9)	−0.0030 (7)	0.0032 (7)	−0.0044 (7)
O2	0.0158 (7)	0.0174 (8)	0.0214 (8)	−0.0014 (6)	0.0052 (6)	−0.0016 (6)
O3	0.0157 (7)	0.0193 (8)	0.0277 (9)	−0.0012 (6)	0.0022 (6)	−0.0038 (6)
N1	0.0182 (9)	0.0182 (9)	0.0167 (9)	0.0019 (7)	0.0070 (7)	−0.0002 (7)
N2	0.0132 (9)	0.0159 (9)	0.0211 (10)	−0.0008 (7)	0.0020 (7)	−0.0023 (7)
C1	0.0225 (12)	0.0194 (11)	0.0303 (13)	−0.0028 (10)	0.0008 (10)	0.0029 (9)
C2	0.0180 (11)	0.0165 (11)	0.0226 (11)	0.0014 (8)	−0.0016 (9)	−0.0004 (8)
C3	0.0249 (12)	0.0198 (11)	0.0171 (11)	0.0046 (9)	−0.0033 (9)	−0.0045 (9)
C4	0.0190 (10)	0.0210 (12)	0.0155 (10)	0.0043 (9)	0.0015 (8)	−0.0021 (8)
C5	0.0169 (10)	0.0169 (10)	0.0137 (10)	0.0016 (8)	0.0016 (8)	0.0023 (8)
C6	0.0183 (10)	0.0175 (11)	0.0189 (10)	0.0013 (9)	0.0011 (8)	0.0005 (8)
C7	0.0133 (9)	0.0139 (10)	0.0168 (10)	0.0014 (8)	0.0012 (8)	0.0007 (8)
C8	0.0128 (10)	0.0176 (10)	0.0155 (10)	0.0027 (8)	0.0008 (8)	0.0006 (8)
C9	0.0160 (10)	0.0179 (11)	0.0171 (10)	0.0005 (9)	0.0017 (8)	0.0031 (8)
C10	0.0144 (10)	0.0138 (10)	0.0169 (10)	0.0016 (8)	0.0003 (8)	0.0007 (8)
C11	0.0163 (10)	0.0159 (11)	0.0141 (10)	−0.0001 (8)	0.0025 (8)	0.0006 (8)
C12	0.0173 (10)	0.0137 (10)	0.0196 (11)	0.0004 (8)	0.0028 (9)	−0.0011 (8)
C13	0.0197 (10)	0.0164 (10)	0.0200 (11)	0.0005 (9)	0.0019 (9)	−0.0015 (8)
C14	0.0204 (11)	0.0154 (10)	0.0225 (11)	0.0022 (8)	0.0035 (9)	0.0023 (8)
C15	0.0199 (11)	0.0148 (10)	0.0209 (11)	0.0004 (8)	0.0053 (9)	−0.0024 (8)
C16	0.0227 (11)	0.0195 (11)	0.0194 (11)	0.0010 (9)	0.0011 (9)	−0.0013 (8)
C17	0.0199 (11)	0.0174 (11)	0.0182 (11)	0.0038 (9)	0.0001 (9)	−0.0015 (8)

### Geometric parameters (Å, °)

**Table:**

O1—C2	1.379 (3)	C7—C10	1.431 (3)
O1—C1	1.427 (3)	C7—C8	1.448 (3)
O2—C10	1.238 (2)	C8—C9	1.403 (3)
O3—C11	1.237 (2)	C9—H9	0.9500
N1—C6	1.344 (3)	C10—C11	1.534 (3)
N1—C5	1.389 (3)	C12—C17	1.526 (3)
N1—H1	0.96 (3)	C12—C13	1.528 (3)
N2—C11	1.331 (3)	C12—H12	1.0000
N2—C12	1.465 (3)	C13—C14	1.530 (3)
N2—H2	0.88 (3)	C13—H13A	0.9900
C1—H1A	0.9800	C13—H13B	0.9900
C1—H1B	0.9800	C14—C15	1.534 (3)
C1—H1C	0.9800	C14—H14A	0.9900
C2—C9	1.379 (3)	C14—H14B	0.9900
C2—C3	1.409 (3)	C15—C16	1.524 (3)
C3—C4	1.375 (3)	C15—H15A	0.9900
C3—H3	0.9500	C15—H15B	0.9900
C4—C5	1.388 (3)	C16—C17	1.532 (3)
C4—H4	0.9500	C16—H16A	0.9900
C5—C8	1.405 (3)	C16—H16B	0.9900
C6—C7	1.399 (3)	C17—H17A	0.9900
C6—H6	0.9500	C17—H17B	0.9900
			
C2—O1—C1	116.63 (17)	O3—C11—N2	123.5 (2)
C6—N1—C5	109.37 (18)	O3—C11—C10	121.94 (18)
C6—N1—H1	122.6 (18)	N2—C11—C10	114.56 (18)
C5—N1—H1	128.1 (18)	N2—C12—C17	112.11 (17)
C11—N2—C12	120.74 (17)	N2—C12—C13	110.36 (18)
C11—N2—H2	119.9 (18)	C17—C12—C13	110.58 (17)
C12—N2—H2	119.2 (18)	N2—C12—H12	107.9
O1—C1—H1A	109.5	C17—C12—H12	107.9
O1—C1—H1B	109.5	C13—C12—H12	107.9
H1A—C1—H1B	109.5	C12—C13—C14	110.68 (18)
O1—C1—H1C	109.5	C12—C13—H13A	109.5
H1A—C1—H1C	109.5	C14—C13—H13A	109.5
H1B—C1—H1C	109.5	C12—C13—H13B	109.5
C9—C2—O1	124.05 (18)	C14—C13—H13B	109.5
C9—C2—C3	121.8 (2)	H13A—C13—H13B	108.1
O1—C2—C3	114.11 (19)	C13—C14—C15	110.64 (18)
C4—C3—C2	121.0 (2)	C13—C14—H14A	109.5
C4—C3—H3	119.5	C15—C14—H14A	109.5
C2—C3—H3	119.5	C13—C14—H14B	109.5
C3—C4—C5	117.46 (19)	C15—C14—H14B	109.5
C3—C4—H4	121.3	H14A—C14—H14B	108.1
C5—C4—H4	121.3	C16—C15—C14	110.60 (18)
C4—C5—N1	129.47 (18)	C16—C15—H15A	109.5
C4—C5—C8	122.22 (19)	C14—C15—H15A	109.5
N1—C5—C8	108.29 (18)	C16—C15—H15B	109.5
N1—C6—C7	109.98 (19)	C14—C15—H15B	109.5
N1—C6—H6	125.0	H15A—C15—H15B	108.1
C7—C6—H6	125.0	C15—C16—C17	111.42 (19)
C6—C7—C10	127.11 (19)	C15—C16—H16A	109.3
C6—C7—C8	106.15 (17)	C17—C16—H16A	109.3
C10—C7—C8	126.63 (17)	C15—C16—H16B	109.3
C9—C8—C5	119.91 (19)	C17—C16—H16B	109.3
C9—C8—C7	133.89 (18)	H16A—C16—H16B	108.0
C5—C8—C7	106.20 (18)	C12—C17—C16	109.81 (17)
C2—C9—C8	117.55 (18)	C12—C17—H17A	109.7
C2—C9—H9	121.2	C16—C17—H17A	109.7
C8—C9—H9	121.2	C12—C17—H17B	109.7
O2—C10—C7	123.40 (19)	C16—C17—H17B	109.7
O2—C10—C11	118.42 (18)	H17A—C17—H17B	108.2
C7—C10—C11	118.15 (17)		
			
C1—O1—C2—C9	−1.3 (3)	C5—C8—C9—C2	−0.7 (3)
C1—O1—C2—C3	177.67 (19)	C7—C8—C9—C2	179.7 (2)
C9—C2—C3—C4	0.6 (3)	C6—C7—C10—O2	174.9 (2)
O1—C2—C3—C4	−178.4 (2)	C8—C7—C10—O2	−0.7 (3)
C2—C3—C4—C5	0.1 (3)	C6—C7—C10—C11	−2.9 (3)
C3—C4—C5—N1	−179.4 (2)	C8—C7—C10—C11	−178.55 (19)
C3—C4—C5—C8	−1.2 (3)	C12—N2—C11—O3	4.1 (3)
C6—N1—C5—C4	178.7 (2)	C12—N2—C11—C10	−175.09 (18)
C6—N1—C5—C8	0.3 (2)	O2—C10—C11—O3	148.5 (2)
C5—N1—C6—C7	−0.2 (2)	C7—C10—C11—O3	−33.6 (3)
N1—C6—C7—C10	−176.2 (2)	O2—C10—C11—N2	−32.4 (3)
N1—C6—C7—C8	0.1 (2)	C7—C10—C11—N2	145.54 (19)
C4—C5—C8—C9	1.5 (3)	C11—N2—C12—C17	−85.5 (2)
N1—C5—C8—C9	−179.95 (19)	C11—N2—C12—C13	150.80 (19)
C4—C5—C8—C7	−178.77 (19)	N2—C12—C13—C14	−177.23 (17)
N1—C5—C8—C7	−0.2 (2)	C17—C12—C13—C14	58.2 (2)
C6—C7—C8—C9	179.8 (2)	C12—C13—C14—C15	−56.8 (2)
C10—C7—C8—C9	−3.9 (4)	C13—C14—C15—C16	55.7 (2)
C6—C7—C8—C5	0.1 (2)	C14—C15—C16—C17	−56.4 (2)
C10—C7—C8—C5	176.4 (2)	N2—C12—C17—C16	178.52 (17)
O1—C2—C9—C8	178.57 (19)	C13—C12—C17—C16	−57.9 (2)
C3—C2—C9—C8	−0.4 (3)	C15—C16—C17—C12	57.4 (2)

### Hydrogen-bond geometry (Å, °)

**Table:**

*D*—H···*A*	*D*—H	H···*A*	*D*···*A*	*D*—H···*A*
N1—H1···O2^i^	0.96 (3)	1.90 (3)	2.840 (2)	164 (3)
N2—H2···O3^ii^	0.88 (3)	2.09 (3)	2.926 (3)	160 (3)

Symmetry codes: (i) *x*+1, *y*, *z*−1; (ii) *x*−1, *y*, *z*.
